# An End‐to‐End Model for Visualization and Highlight of Alzheimer's Disease Progression

**DOI:** 10.1002/alz.090206

**Published:** 2025-01-09

**Authors:** Juexin Zhang, Ying Weng, Weizhong Xiao

**Affiliations:** ^1^ The Universit of Nottingham Ningbo China, Ningbo, Zhejiang China; ^2^ University of Nottingham Ningbo China, Ningbo, Zhejiang China; ^3^ Peking University Third Hospital, Beijing China

## Abstract

**Background:**

Subtle cognitive decline in the prodromal stages of Alzheimer's disease (AD), encompassing both significant memory concerns (SMC) and mild cognitive impairment (MCI), presents a crucial diagnostic hurdle. This challenge stems from the inherent difficulty in both detecting the nuanced neurocognitive aberrations and visualizing the corresponding brain alterations, particularly within the hippocampus, on conventional magnetic resonance imaging (MRI). Consequently, current clinical diagnostic procedures remain arduous and necessitate substantial clinical expertise. Considering these limitations, the development of automated deep learning‐based methods for visualizing early‐stage AD‐related brain changes holds immense promise.

**Method:**

We propose a generative model capable to synthesize high quality MRIs for both retroactive and predictive visualization of a given MRI. Furthermore, our model also indicates the morphological changes of a given MRI. Our model is comprised of three interconnected components: a classifier, a generative model, and a morphological feature identifier. Initially, a convolutional neural network (CNN) classifier categorizes the input MRI into its corresponding stage within the disease progression spectrum. Subsequently, the generative model, constructed to capture the nuances of disease‐related morphological evolution, synthesizes MRIs that simulate the brain's structural configuration at other potential stages of the disease. Finally, the morphological feature identifier, through a process of automated segmentation, delineates areas exhibiting significant morphological deviations and highlights those regions deemed most pertinent for further clinical evaluation.

**Result:**

We employed T1‐weighted MRIs from the Alzheimer’s Disease Neuroimaging Initiative (ADNI) public dataset to test our model. Our synthetic MR images achieved high visual fidelity, as well as indistinguishable from real images for human observers, and yielded high peak signal‐to‐noise ratio (PSNR) and structural similarity index measure (SSIM) scores. Moreover, our model can precisely highlight morphological deviations.

**Conclusion:**

To facilitate diagnosis and intervention in early AD, we put forward a generative model to synthesize other stages of a given MRI. This allows clinicians to visualize potential disease progression pathways and clearly observe morphological changes in vulnerable brain regions. Our model can also highlight key anatomical alterations to help AD analysis and offer valuable insights into promise for early AD management.

